# Water-soluble polyphenol-rich clove extract lowers pre- and post-prandial blood glucose levels in healthy and prediabetic volunteers: an open label pilot study

**DOI:** 10.1186/s12906-019-2507-7

**Published:** 2019-05-07

**Authors:** Ratheesh Mohan, Svenia Jose, Johannah Mulakkal, Darla Karpinsky-Semper, Andrew G. Swick, I. M. Krishnakumar

**Affiliations:** 1 Department of Biochemistry, St. Thomas College, Pala, Kerala India; 2 R&D center, Akay Flavours & Aromatics Pvt Ltd, Cochin, India; 3 Life Extension, Ft. Lauderdale, FL 33308 USA

## Abstract

**Background/objectives:**

Type 2 diabetes (T2D) is a global pandemic, and contributes significantly to the increasing incidence of conditions such as cardiovascular disease (CVD). Postprandial plasma glucose measured 2-h after the start of a meal is a good indicator of the overall status of glucose homeostasis. Clove (*Syzygium aromaticum L.*) and its essential oils (eugenol and acetyl eugenol) have been shown in preclinical studies to modulate pathways involved in glucose homeostasis. In addition, a water-soluble polyphenolic extract of unopened clove buds was recently shown to benefit liver function and redox status. Therefore, we conducted an open-label pilot study to test whether this polyphenolic clove extract (PCE) could influence glucose metabolism.

**Methods:**

We evaluated the effect of PCE supplementation (250 mg once daily for 30 days) on preprandial glucose levels and 2-h postprandial glucose levels in 13 otherwise healthy volunteers who were stratified into two groups according to their initial preprandial glucose levels: Group I (*n* = 7) ≤100 mg/dL, Group II (*n* = 6) – between 101 and 125 mg/dL. In an effort to elucidate the molecular mechanisms of PCE action, we tested in vitro the effects of PCE on glucose uptake, hepatocyte glucose production, and carbohydrate hydrolyzing enzymes.

**Results:**

At day 12 of supplementation, we observed statistically significant reductions in mean postprandial glucose levels in both groups [(Group I: Initial - Day 12 PPG = 13.29 mg/dL, 95% CI: 3.329–23.24) (Group II: Initial – Day 12 PPG = 16.67 mg/dL, 95% CI: 4.687–28.65, *P* = 0.0159)], which continued through study completion at day 30. PCE supplementation significantly decreased mean preprandial glucose levels only in Group II at Days 24 (Initial – Day 24 = 13.00 mg/dL, 95% CI: 1.407–24.59, *P* = 0.0345) and 30 (Initial – Day 30 = 13.67 mg/dL, 95% CI: 5.766–21.57, *P* = 0.0067). In cell-based assays, PCE enhanced glucose uptake in L6 myocytes and inhibited hepatocyte glucose production HepG2 cells. In cell-free assays, PCE inhibited α-amylase activity and α-glucosidase activity.

**Conclusions:**

These findings underscore the therapeutic utility of PCE for maintaining healthy glucose metabolism and warrant further larger-scale clinical trials.

**Trial registration:**

This trial was retrospectively registered in the ISRCTN registry on September 29, 2018 (ISRCTN15680985).

## Background

Type 2 diabetes is a tremendous public health issue. More than 75% of the US population over 65 years of age has some degree glucose homeostasis dysfunction [1]. In 2014, adults 25 to 44 years of age were more than twice as likely to have diabetes and be overweight or obese than in 1989 [2]. Factors driving this metabolic syndrome pandemic include alterations in diet and reduced physical activity [3]. Diets have shifted from nutrient-dense (fresh vegetables and fruits, and unrefined grains) to energy-rich but nutrient-poor (refined grains, sugars and fats) [4]. Numerous population studies across the globe have demonstrated that polyphenol intake has an inverse relationship with disease incidence [5–7]. And, diets low in polyphenols are associated with an increase in T2DM incidence [8–10]. PCE is a water-soluble standardized extract of unopened clove (*Syzygium aromaticum L.*) buds that contains a minimum of 30% total polyphenols [11–13]. HPLC analysis of PCE revealed the presence of gallic acid, ellagic acid, catechin, quercetin, chlorogenic acid, and eugenol [12].

The liver plays a major role in maintaining ideal levels of glucose throughout the body by regulating de novo glucose production (gluconeogenesis) and glycogen breakdown (glycogenolysis) [14]. Dysregulated and exaggerated hepatic glucose production (HGP) can result in poor clinical outcomes and is a major contributor to impaired glucose homeostasis and type 2 diabetes [14]. Supporting the idea that insulin resistance originates in liver tissue, a preclinical model of diet-induced obesity demonstrated that insulin resistance was first detected in the liver, then in white adipose tissue [15].

Our earlier preclinical and clinical studies demonstrated beneficial effects of PCE on liver function, antioxidant status, and metabolism of alcohol [11, 12, 16]. We thus hypothesized that, since the liver plays a crucial role in the pathogenesis of insulin resistance and diabetes, PCE could have a beneficial effect on glucose metabolism as well. We tested the effects that daily supplementation with PCE has on preprandial and postprandial blood glucose levels in an open-label pilot study. We also used a series of in vitro assays to elucidate PCE’s mechanism of action on glucose metabolism.

## Materials and methods

### Clinical study design

The clinical study protocol was reviewed approved by an independent ethics committee (Reg. No: ECR/184/Indt/KA/2014). We evaluated the effect of PCE (250 mg × 1; hard shell, two-piece gelatin capsule) on the preprandial and postprandial plasma glucose levels of 13 volunteers (10 males and 3 females, aged 25 to 35 years). We grouped the subjects according to their initial plasma glucose levels. Group I had pre-lunch plasma glucose levels ≤100 mg/dL; Group II had levels between 101 and 125 mg/dL. We asked all subjects to complete their breakfast by 8 AM and not to consume any food or snacks for another 4 h (until 12 noon). The subjects had *ad libitum *water access. We measured subjects’ blood sugar levels with a digital glucometer at 12:00 PM and marked this sample as the “preprandial” plasma glucose level. Subjects then consumed a typical south-Indian lunch with rice, vegetable curry with meat or fish, and water within 30 min. We provided one capsule of PCE to each subject immediately after lunch. After 2 h, we measured subjects’ plasma glucose levels again and marked the sample as “postprandial.” This routine continued for 30 days, and we recorded preprandial and postprandial glucose levels on days 1, 12, 24 and 30. Day 1 was “without PCE” and it is taken as the baseline value. Throughout the study and analysis, no investigators were blinded.

### Clinical study participants

All volunteers provided written informed consent before the study. The subjects were generally healthy and did not take any regular medication. Physical activity levels were similar among the participants. We advised the participants to follow their normal south-Indian food pattern, which typically consists of: breakfast made of rice (having an approximate nutritional composition of fat: 22 to 24%, carbohydrates: 35 to 40%, protein: 27 to 30%; energy 400 to 500 cal), lunch with rice and vegetable/non-vegetable curries (having an approximate nutritional composition of fat: 25 to 30%, carbohydrates: 35 to 42%, protein: 32 to 35%; energy 500 to 580 cal), and dinner with similar nutritional compositions to lunch.

### Clinical study material

As described previously, we prepared PCE by the hydro-ethanolic extraction of dried clove buds followed by low temperature evaporation and spray drying to free flowing powder with not less than 30% polyphenols as gallic acid equivalent [11–13].

### Glucose uptake assay

We conducted the glucose uptake assay according to the protocol described by Yap et al., 2007 [17]. We purchased L6 rat myoblast cells from NCCS Pune and maintained them in Dulbecco’s modified eagles media (Sigma Aldrich, USA) supplemented with 10% FBS (Invitrogen) and grew them to 80% confluency at 37 °C in 5% CO_2_ in a humidified atmosphere in a CO_2_ incubator (NBS, Eppendorf, Germany). We detached cells with trypsin [500 μL of 0.025% Trypsin in PBS/0.5 mM EDTA solution (Invitrogen)] for 2 min then passaged them to T-75 flasks in complete aseptic conditions. We then subcultured the cells to a 24-well plate. After the cells attained 80% confluency, we kept them in DMEM without glucose for 24 h. We then added extracts to grown cells at a final concentration of 25 μg, 50 μg, and 100 μg from a stock of 1 mg/mL and incubated them for 24 h in DMEM containing 300 mM glucose. We also maintained an untreated control with high glucose. After incubation, we isolated the cells by spinning them at 6000 rpm for 10 min. We then discarded the supernatant and added 200 μL of cell lysis buffer (1 M Tris HCl, 0.25 M EDTA, 2 M NaCl, 0.5% Triton). The incubation period was 30 min at 37 °C, and we estimated the glucose uptake using a high-sensitivity glucose oxidase kit (Glucose oxidase kit, Erba). We repeated all experiments in triplicates and used mean average to calculate percent glucose uptake relative to untreated controls.

### Endogenous glucose production assay

We conducted the glucose production assay according to the protocol described by Caton et al., 2010 [18]. We purchased HepG2 cells from NCCS Pune and maintained them in Dulbecco’s modified eagles media (Sigma Aldrich) supplemented with 10% FBS (Invitrogen) and grew them to 80% confluency at 37 °C in 5% CO_2_. We next subcultured the cells to a 24-well plate. After the cells attained 80% confluency, we kept them in DMEM without glucose and treated them with PCE at final concentrations of 25 μg/mL, 50 μg/mL, or 100 μg/mL for 30 min and incubated them for 6 h after adding 100 nM glucagon. We also maintained controls (i.e., without PCE) under similar conditions. After incubation, we isolated the cells by spinning them at 6000 rpm for 10 min. We then discarded the supernatant and added 200 μL of cell lysis buffer (1 M Tris HCl, 0.25 M EDTA, 2 M NaCl, 0.5% Triton). The incubation period was 30 min at 37 °C, and we estimated the glucose content using a glucose-kit method (Erba Mannheim, Germany) and read absorbance at 505 nm (Agilent, USA). We repeated all experiments in triplicates and used the mean average to calculate the percent glucose production relative to untreated controls.

### α-Glucosidase inhibition assay

We measured α-glucosidase activity in a cell-free assay by determining the reducing sugars formed upon hydrolysis of sucrose by α-glucosidase enzyme. We determined the effect of PCE on α-glucosidase activity according to the method described by Matsui and colleagues [19], with slight modifications. Briefly, we pre-incubated 1 mg α-glucosidase protein (Sigma Aldrich) with different concentrations of PCE for 5 min at 37 °C. Then, we added the substrate sucrose (37 mM), and brought the final reaction mixture to 1 mL with 0.1 M phosphate buffer (pH 7.2). We incubated the reaction mixture for 30 min at 37 °C then stopped the reaction by incubating the mixture in a boiling water bath for 2 min. We maintained a tube treated with phosphate buffer and enzyme as a control. Next, we added 250 μL of glucose reagent (Glucose oxidase kit, Erba) to each tube and incubated them for 10 min before measuring absorbance at 510 nm. The α-glucosidase inhibitory activity is expressed as percentage of inhibition relative to untreated controls.

### α-Amalyase inhibition assay

We performed this assay according to the method developed by Bernfeld in 1955 [20]. We pre-incubated different concentrations of PCE with 25 μL of porcine α-amylase (0.5 mg/mL) at 25 °C for 10 min. After pre-incubation, we added 25 μL of 0.5% starch solution in 25 mM phosphate buffer (pH 6.9). We then incubated the reaction mixtures at 25 °C for 10 min. We maintained enzyme preparations treated with PBS under the same conditions as controls. We stopped the reaction with 50 μL of 96 mM 3,5-dinitrosalicylic acid color reagent. We then incubated the micro-plate in a boiling water bath for 5 min and cooled it to room temperature. We measured absorbance at 540 nm using a microplate reader (Erba, Lisascan). The α-amalyase inhibitory activity is expressed as percentage of inhibition relative to untreated controls.

### Statistical analysis

We used GraphPad Prism 5.0 software to analyze all of the data. For the pilot study, we used a paired, two-tailed t-test to detect significant differences between glucose levels recorded at each time point and those collected at baseline. We calculated mean differences, 95% confidence intervals and *P* values, which are tabulated in Table 1. For in vitro experiments, we used the non-linear regression log (inhibitor) vs response equation with variable slope to calculate the IC_50_ values. We tabulated standard error, 95% confidence intervals, and goodness of curve fitting (R^2^) in Table 2.

**Table 1:** Pilot study statistics

Comparison	Δ Plasma Glucose (mg/dL) [Initial – Day]	95% Confidence Interval	*P* Value
Group I Preprandial			
Initial vs. Day 12	1.286	− 5.752 - 8.324	0.6705
Initial vs. Day 24	2.000	−3.150 - 7.150	0.3786
Initial vs. Day 30	4.857	−2.032 - 11.75	0.1352
Group II Preprandial			
Initial vs. Day 12	0.5000	−6.764 - 7.764	0.8665
Initial vs. Day 24	13.00	1.407–24.59	**0.0345**
Initial vs. Day 30	13.67	5.766–21.57	**0.0067**
Group I Postprandial			
Initial vs. Day 12	13.29	3.329–23.24	**0.0171**
Initial vs. Day 24	17.29	0.9953–33.58	**0.0409**
Initial vs. Day 30	27.00	3.378–50.62	**0.0313**
Group II Postprandial			
Initial vs. Day 12	16.67	4.687–28.65	**0.0159**
Initial vs. Day 24	30.50	3.726–57.27	**0.0327**
Initial vs. Day 30	40.33	15.23–65.43	**0.0091**

We compared preprandial and postprandial plasma glucose levels taken at each time point to initial values and calculated statistical significance using a paired t-test in GraphPad Prism 5.0 software. Mean change in plasma glucose, 95% confidence interval, and *P* value are shown for each time point as compared to initial values, with *P* ≤ 0.05 in bold text

**Table 2:** In vitro study statistics

IC_50_	Standard Error	95% Confidence Interval	R^2^
Glucose Production			
63.80 μg/mL	0.0632	45.22–90.01	0.7192
α-Glucosidase Activity			
PCE			
60.84 mg/mL	0.0283	52.84–70.05	0.9523
Acarbose			
40.28 mg/mL	0.0276	35.00–46.21	0.9568
α-Amylase Activity			
51.63 μg/mL	13.29	45.39–58.71	0.9615

For the inhibitory action that PCE has on glucose production and carbohydrate hydrolyzing enzymes, we calculated the IC_50_ values using the log (inhibitor) vs. response equation with variable slope in GraphPad Prism 5.0 software. Standard error, 95% confidence intervals, and goodness of curve fitting (R^2^) are shown

## Results

**Fig. 1:**
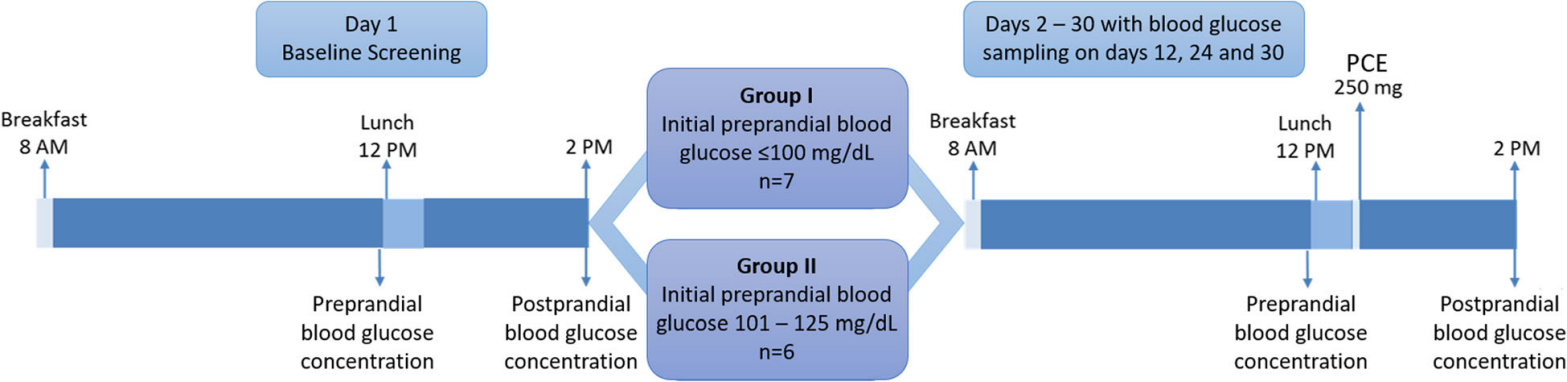
Pilot study design. We measured preprandial blood glucose4 h after breakfast. During the time-period between breakfast and lunch, we instructed volunteers to refrain from consuming any food or beverage, except water, which was provided *ad libitum*. Lunch consisted of a typical south Indian meal of rice, vegetable curry with meat or fish to be consumed within 30 m. We measured postprandial glucose 2 h after the start of lunch. On the initial day of the study (Day 1), volunteers followed this routine and we categorized the patients into two groups according to their preprandial glucose levels. Then, for the next 30 days, volunteers consumed PCE (250 mg) 5 min after lunch. We assessed blood glucose on days 12, 24, and 30

Figure 1 shows the open-label trial design. On the initial day of the trial, 13 subjects were assigned to one of two groups based on their preprandial blood glucose levels tested just before lunch 4 h after breakfast (Fig. 1). Group I consisted of 7 individuals with preprandial blood glucose ≤100 mg/dL, and Group II was comprised of individuals with preprandial glucose between 101 and 125 mg/dL (Fig. 1). The study material, PCE, was supplied in one gelatin capsule at 250 mg/capsule. On trial days 2–30, subjects took one PCE capsule immediately after finishing lunch. Preprandial and 2-h postprandial glucose measurements were taken on days 1, 12, 24, and 30 of the study.

**Fig. 2:**
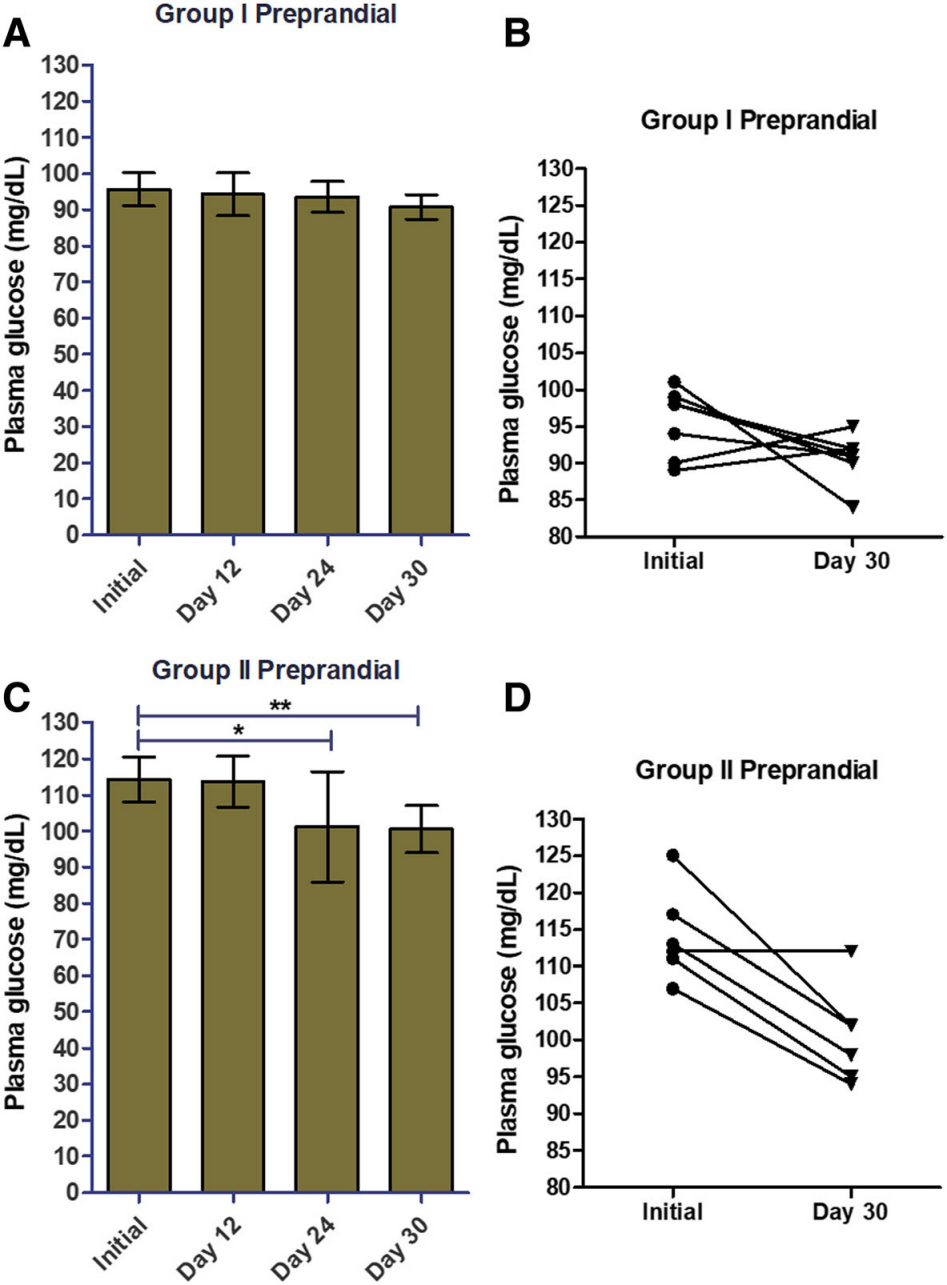
PCE supplementation reduces preprandial glucose levels in the prediabetes group, but not in the group with blood glucose already within normal range. On the initial day of the study**,** we divided individuals into two groups based on their preprandial (before lunch) plasma glucose levels: **a**-**b**, Group I, ≤100 mg/dL; **c**-**d**, Group II, 101–125 mg/dL. Pre-prandial plasma glucose levels were measured and recorded on days 1 (initial), 12, 24, and 30 (**a**, **c**). We plotted individual study subject plasma glucose levels for the initial day and day 30 of the study (**b**, **d**). Data shown are mean ± SD for **a** and **c**. We calculated statistical significance (*, *p* ≤ 0.05; **, *p* ≤ 0.01) by comparing glucose levels measured at each time point to the initial value using a two-tailed t-test in GraphPad Prism 5.0 software

For Group I, PCE supplementation did not alter preprandial glucose levels throughout the duration of the study (Fig. 2a-b; Table 1). However, in Group II significant decreases in preprandial glucose levels manifested on day 24 and continued to study completion (Fig. 2c-d; Table 1). The mean preprandial glucose level in Group II fell by 12% from baseline 114.2 ± 2.54 mg/dL [mean ± SD] to day 30; 100.5 ± 2.68 mg/dL [mean ± SD] (Fig. 2d; Table 1). These results indicate that PCE is unlikely to cause hypoglycemia as preprandial glucose was not affected in individuals whose levels were initially within a normal range of less than 100 mg/dL (Fig. 2a-b; Table 1), but decreased in individuals who displayed tendencies of abnormal glucose homeostasis (Fig. 2c-d; Table 1). Since PCE was administered once daily after lunch, the reduction in preprandial glucose levels in Group II suggests that PCE is effective at maintaining normal blood glucose levels throughout the day in individuals with tendencies of abnormal glucose homeostasis. Taken together, these data demonstrate the efficacy of once daily PCE supplementation at managing glucose homeostasis throughout the day.

**Fig. 3:**
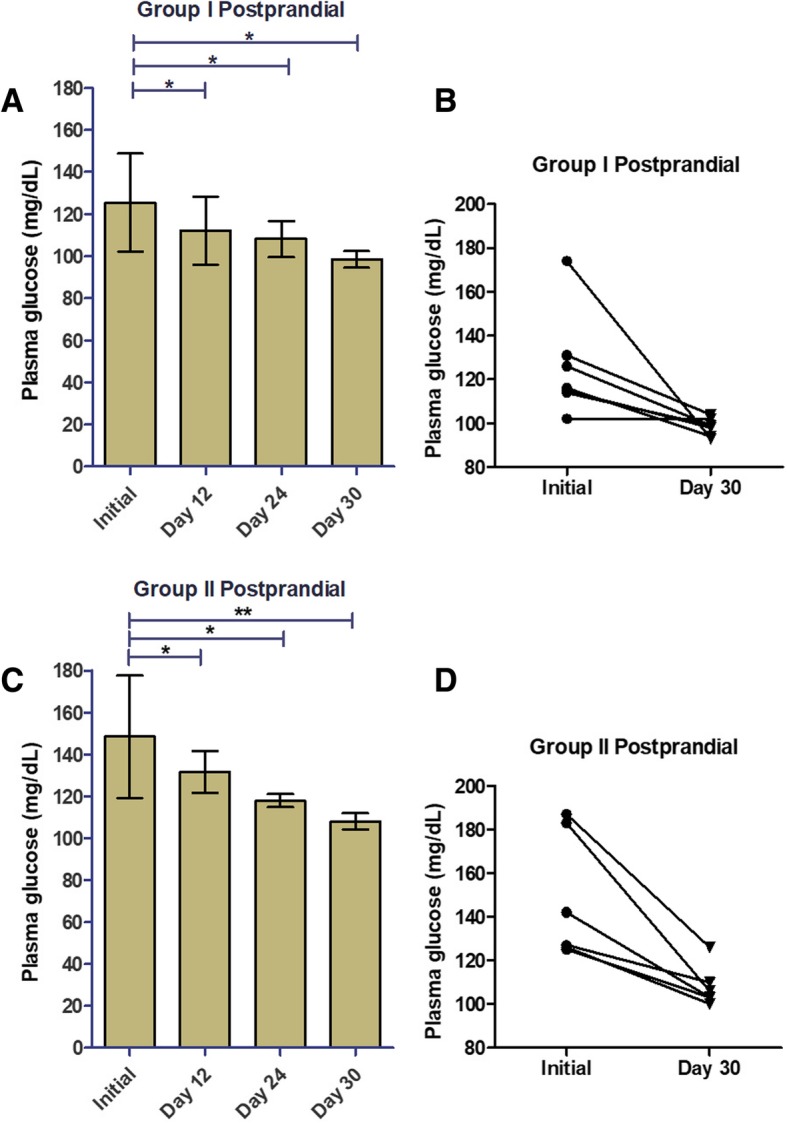
In both normal and prediabetic individuals, PCE supplementation decreased postprandial blood glucose. After the initial day of the study, subjects consumed one PCE capsule immediately after finishing lunch each day for the duration of the study and postprandial glucose was measured 2 h after the start of lunch. Postprandial plasma glucose levels were measured and recorded on days 1 (initial), 12, 24, and 30 (**a**, **c**). We plotted individual study subject plasma glucose levels for the initial day and day 30 of the study (**b**, **d**). Data shown are mean ± SD for **a** and **c**. We calculated statistical significance (*, *p* ≤ 0.05; **, *p* ≤ 0.01) by comparing glucose levels measured at each time point to the initial value using a two-tailed t-test in GraphPad Prism 5.0 software

PCE supplementation controlled postprandial glucose levels in both groups (Fig. 3). For Groups I and II, significant reductions in 2-h post-prandial glucose levels were seen at the earliest time point, day 12, and continued throughout the study (Fig. 3a and c; Table 1). In Group I, the postprandial baseline value was 125.4 ± 8.82 mg/dL (mean ± SD), and by day 30 PCE supplementation significantly reduced this value by 21.5% to 98.43 ± 1.49 mg/dL (mean ± SD) (Fig. 3b; Table 1). There was a more pronounced effect on postprandial glucose in Group II: PCE significantly reduced postprandial glucose by 27.2% from 148.3 ± 11.88 mg/dL (mean ± SD) at baseline to 108 ± 3.86 mg/dL (mean ± SD) at day 30 (Fig. 3d; Table 1). These results clearly support the rationale for once daily PCE supplementation to promote healthy glucose metabolism, in particular, post-prandial glucose.

**Fig. 4:**
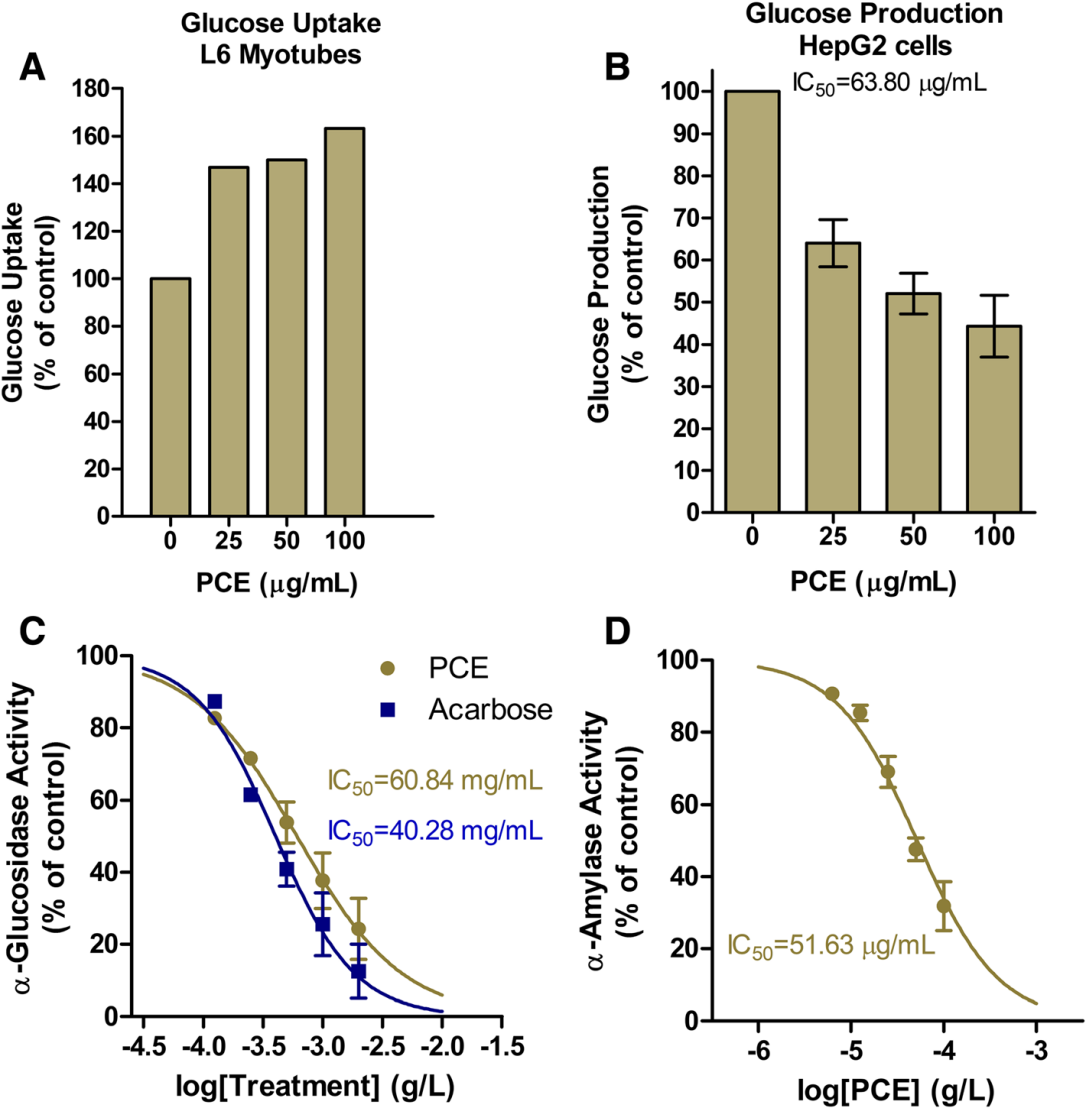
In vitro effects of PCE on key aspects of glucose homeostasis. (**a**) Glucose uptake in L6 myotubes in the absence or presence of increasing concentrations of PCE. (**b**) Hep2G cell endogenous glucose production in the absence or presence of increasing concentrations of PCE. (**c**-**d**) Concentration-response curve for PCE () and Acarbose () mediated inhibition of α-glucosidase (**c**) and α-amalyase enzyme activity (**d**) in cell-free systems. All data are represented as mean ± SD percent activity relative to untreated controls. We derived IC_50_ values and fit curves using a non-liner inhibitory concentration-response equation with variable slope in GraphPad Prism 5.0 software

Next, we carried out in vitro assays to elucidate the molecular mechanism(s) by which PCE lowers glucose (Fig. 4). In this first set of in vitro experiments, we assessed glucose uptake in L6 myotubes in the absence or presence of increasing PCE concentrations. We found that PCE dose-dependently enhanced glucose uptake in this cell model (Fig. 4a). PCE concentrations of 25, 50 and 100 μg/mL increased glucose uptake by 46.97, 50.02 and 63.36% over untreated controls, respectively (Fig. 4a). This finding suggests that PCE could be increasing insulin sensitivity to lower plasma glucose as observed in Figs. 2 and 3.

Then, we investigated the effects of PCE on endogenous glucose production in hepatocytes, which is arguably one of the most important sites for controlling postprandial plasma glucose. Briefly, we incubated glucose-starved HepG2 cells with glucagon (to stimulate gluconeogenesis and glycogenolysis) in the presence or absence of increasing concentrations of PCE. In a concentration-dependent manner, PCE inhibited hepatocyte glucose production with an IC_50_ of 63.8 μg/mL (Fig. 4; Table 2). This finding is consistent with the results of a previous study that identified a eugenol-depleted hydroethanolic clove extract inhibited glycogen phosphorylase b (IC_50_ = 0.86 μg/mL), the enzyme responsible for converting liver glycogen to glucose [21]. This PCE mechanism likely accounts for the marked improvements in postprandial plasma glucose observed in our study participants (Figs. 2c-d; 3c-d), but especially Group II (Fig. 3c-d).

In the next set of in vitro experiments, we tested the ability of PCE to inhibit key carbohydrate hydrolyzing enzymes, α-amylase and α-glucosidase, in cell-free assays. PCE dose-dependently inhibited both enzymes (Fig. 4c-d; Table 2). The efficacy of PCE at inhibiting α-glucosidase activity with an IC_50_ of 60.84 mg/mL was similar to that of the antidiabetic drug acarbose, which has an IC_50_ of 40.28 mg/mL at this enzyme (Fig. 4c; Table 2). PCE was more efficacious at inhibiting α-amylase with an IC_50_ 42.87 μg/mL (Fig. 4d; Table 2).

## Discussion

Although fasting plasma glucose is the traditional standard for diabetes screening, impaired glucose tolerance (IGT) may be present in individuals with normal fasting glucose. And IGT may have a slightly stronger association with cardiovascular risk than IFG alone [22]. Hemoglobin A_1c_ (HbA_1c_) is a measure of the degree to which hemoglobin is glycosylated in erythrocytes and is expressed as a percentage of total hemoglobin concentration [23]. It reflects the exposure of erythrocytes to glucose in an irreversible and time- and concentration-dependent manner [23]. HbA_1c_ levels provide an indication of the average blood glucose concentration during the preceding 2–3 months, incorporating both pre- and post-prandial glycemia. While HbA_1c_ is an indicator of long-term glucose homeostasis, it may not reflect the magnitude of acute glucose spikes during the postprandial period [23]. Moreover, clinically significant inter-subject variability exists and HbA_1c_ measurements inadequately reflect actual glycemic control for many patients [24].

Several studies have highlighted the correlation between postprandial blood glucose and cardiovascular events and mortality [25–32]. In aged type 2 diabetics, exaggerated PPG excursions are associated with impaired cognitive function [33]. Therapies that target PPG excursions have demonstrated their utility in the clinic [34]. Thus, interventions that delay or prevent postprandial hyperglycemia could be significantly useful in curbing the impaired glucose homeostasis pandemic.

Glucose metabolism is a tightly regulated process. After eating, α-amylase and α-glucosidase enzymes digest carbohydrates into simple sugars. This excursion of glucose into the bloodstream stimulates secretion of insulin from pancreatic β-cells, while attenuating secretion of glucagon from α-cells. Insulin signaling promotes glucose uptake into peripheral sites in the body where it serves as an energy source. Skeletal muscle and liver are particularly important sites of energy storage. Excess glucose, stored as glycogen, in liver and skeletal muscle is utilized during periods of vigorous activity or fasting via glucagon signaling. Once glycogen stores in muscle and liver reach full capacity, excess glucose is then stored as fat. Thus, maintaining optimal glucose metabolism hinges upon insulin secretion from the pancreas and sensitivity of peripheral tissue to insulin.

The marked decrease in postprandial glucose levels we observed in the pilot study could be attributed to a decrease in hepatic glucose production. Our in vitro data supports this hypothesis as PCE inhibited hepatocyte glucose production with an IC_50_ of 63.8 μg/mL (Fig. 4b; Table 2). In another study using a cell-free assay, a polyphenol-rich clove extract inhibited glycogen phosphorylase b (IC_50_ = 0.86 μg/mL), the enzyme responsible for converting liver glycogen to glucose [21]. One can speculate that our PCE may be inhibiting glycogen phosphorylase b, although further testing is needed to confirm this hypothesis.

## Conclusion

Taken together, the results of the present study indicate that PCE potentially works through multiple molecular mechanisms to control glucose homeostasis. This is apparent in the clinical study results where significant changes in postprandial blood glucose were observed at the first time point in both groups (Fig. 3), but preprandial blood glucose changes were not recorded until later in the study and only in the group that had higher baseline levels, Group II (Fig. 2). The early changes in postprandial blood glucose can likely be attributed to PCE’s inhibitory effects on α-glucosidase and α-amylase enzymes. It stands to reason that the continued improvement in postprandial blood glucose over time in both groups could involve increased insulin sensitivity and decreased hepatic glucose production. Similarly, the full effects of metformin on blood sugar can take up to 4–6 weeks to be seen, and metformin increases insulin sensitivity in liver and muscle [35]. Its action on liver results in decreased hepatic glucose production, which is arguably one of the most important sites for controlling plasma glucose. These positive results of PCE on glucose homeostasis control warrant further investigation into its therapeutic utility as a treatment option delaying and/or preventing the transition from pre-diabetes to type 2 diabetes.

## Abbreviations

CVD: Cardiovascular disease
HbA_1c_: Hemoglobin A_1c_
HGP: Hepatic glucose production
IGT: Impaired glucose tolerance
PCE: Polyphenolic clove extract
PPG: Postprandial glucose
T2D: Type 2 diabetes mellitus
